# Contribution of Pro-inflammatory Cytokine Signaling within Midbrain Periaqueductal Gray to Pain Sensitivity in Parkinson’s Disease *via* GABAergic Pathway

**DOI:** 10.3389/fneur.2016.00104

**Published:** 2016-07-25

**Authors:** Xianbo Zhuang, Yanxiu Chen, Xianpeng Zhuang, Tuanzhi Chen, Tao Xing, Weifei Wang, Xiafeng Yang

**Affiliations:** ^1^Department of Neurology, Liaocheng People’s Hospital, Liaocheng, China; ^2^Department of CT, Liaocheng Fourth People’s Hospital, Liaocheng, China; ^3^Department of Neurosurgery, Liaocheng People’s Hospital, Liaocheng, China

**Keywords:** mechanical sensitivity, thermal sensitivity, neurodegeneration, central inhibition

## Abstract

**Background/aims:**

Hypersensitive pain response is often observed in patients with Parkinson’s disease (PD); however, the mechanisms responsible for hyperalgesia are not well understood. Chronic neuroinflammation is one of the hallmarks of PD pathophysiology. Since the midbrain periaqueductal gray (PAG) is an important component of the descending inhibitory pathway controlling on central pain transmission, we examined the role for pro-inflammatory cytokines (PICs) system of PAG in regulating exaggerated pain evoked by PD.

**Methods:**

We used a rat model of PD to perform the experimental protocols. PD was induced by microinjection of 6-hydroxydopamine to lesion the left medial forebrain bundle. Pain responses to mechanical and thermal stimulation were first examined in control rats and PD rats. Then, ELISA and Western Blot analysis were used to determine PIC levels and their receptors expression.

**Results:**

Protein expression of IL-1β, IL-6, and TNF-α receptors (namely, IL-1R, IL-6R, and TNFR subtype TNFR1) in the plasma membrane PAG of PD rats was upregulated, whereas the total expression of PIC receptors was not significantly altered. The ratio of membrane protein and total protein (IL-1R, IL-6R, and TNFR1) was 1.48 ± 0.15, 1.59 ± 0.18, and 1.67 ± 0.16 in PAG of PD rats (*P* < 0.05 vs. their respective controls). This was accompanied with increases of PICs of PAG and decreases of GABA (623 ± 21 ng/mg in control rats and 418 ± 18 ng/mg in PD rats; *P* < 0.05 vs. control rats) and withdrawal thresholds to mechanical and thermal stimuli. Our data further showed that the concentrations of GABA and withdrawal thresholds were largely restored by blocking those PIC receptors in PAG of PD rats. Stimulation of GABA receptors in PAG of PD rats also blunted a decrease in withdrawal thresholds.

**Conclusion:**

Our data suggest that upregulation of the membrane PIC receptor in the PAG of PD rats is likely to impair the descending inhibitory pathways in regulating pain transmission and thereby plays a role in the development of hypersensitive pain response in PD.

## Introduction

Parkinson’s disease (PD) is characterized by the loss of central dopaminergic (DA) neurons and the presence of α-synuclein-containing aggregates in the substantia nigra pars compacta (1). Notably, epidemiological studies indicate that a high frequency of hypersensitive pain is presented in PD patients (2, 3). Also, behavioral studies show that sensitivity to pain is increased in patients (4, 5). Central nervous mechanisms are considered to play an important role in processing abnormalities in pain response in PD patients (6). Non-DA neurotransmission has been implicated in influencing descending pain pathways at central levels (6). Nonetheless, treatment options for these abnormal pain sensations have been limited, partly because of our poor understanding of the neural mechanisms responsible for PD-induced pain. Thus, it is significant to investigate the pathophysiology of pain abnormalities in patients with PD.

As a component of the descending pain modulatory network, the midbrain periaqueductal gray (PAG) has an inhibitory or excitatory control on pain transmission *via* the rostral ventromedial medulla, projecting to the spinal and medullary dorsal horn (7–9). Accordingly, in the present study, we examined the underlying mechanisms by which the changes in neural substrate activity in the PAG are engaged in PD-induced pain.

Chronic neuroinflammation is one of the hallmarks in PD (10, 11). Studies in human PD patients and animal models of experimental PD show that activation of glial cells and elevation of pro-inflammatory cytokines (PICs, i.e., IL-1β, IL-6, and TNF-α) levels are common features of the PD brain (12–15). Chronic release of PICs by stimulated astrocytes and microglia leads to the exacerbation of DA neuron degeneration in the substantia nigra pars compacta (13, 15). Also, peripheral immune system is involved in the pathogenesis of PD. Infiltration and accumulated immune cells from the periphery are identified in and around the affected brain regions of PD patients (13, 15). Moreover, inflammatory processes have been suggested as promising interventional targets for PD and even other neurodegenerative diseases (12, 13, 14, 15). A better understanding of the role of inflammation in PD will provide new insights into the pathological processes and help to establish effective therapeutic strategies.

GABA is a main inhibitory neurotransmitter in the central nerve system in control of neuronal excitability. After GABA release from presynaptic terminals, GABA transporters play a role in regulating a rapid removal of extracellular GABA (16, 17), which thereby leads to ending of inhibitory synaptic transmission. Thus, this mechanism is responsible for GABA spillover to neighboring synapses (16, 18) and GABA homeostasis (16, 19). In contrast, under certain pathological and physiological conditions, the abnormal levels of GABA are observed (20, 21). A recent study suggests that PIC pathways are upregulated in the brain of rats with excitatory neuronal activities, and this alters expression of GABA *via* IL-1β and TNF-α receptors (22).

Therefore, in this study, we determined the levels of IL-1β, IL-6, and TNF-α and their receptors expression in PAG tissues of PD rats and control rats. Also, we examined if PIC pathways are involved in pain response in PD *via* the descending pain modulatory mechanisms. We hypothesized that protein expression of PIC receptors is upregulated in the PAG of PD rats, and blocking PIC receptors in the PAG attenuates amplified pain responses in PD *via* GABAergic inhibitory pathways.

## Materials and Methods

### Animals

The Research Administration Animal Committee of Liaocheng People’s Hospital approved the procedures outlined in this study (#AS0122015), which were compliant with the guidelines of the International Association for the Study of Pain. One hundred twenty-one male Sprague-Dawley rats weighing 150–200 g were used in this study. The rats were housed in individual cages with free access to food and water and were kept in a temperature-controlled room (25°C) on a 12/12 h light/dark cycle.

### Induction of PD by 6-Hydroxydopamine Lesions

Animals were anesthetized by sodium pentobarbital (60 mg/kg, i.p., Sigma Co.) and then placed in a David Kopf stereotaxic instrument. Injections of 6-hydroxydopamine (6-OHDA) (7 μg/2 μl/each location, dissolved in saline containing 0.02% ascorbic acid) were made at two locations into the left medial forebrain bundle. Stereotaxic coordinates for the lesions were 3.3 mm rostral to the interaural line, 1.4 mm left of the midline, and 6.5 and 6.8 mm (2 μl each location) ventral to the dural surface. The 6-OHDA solution was administered through a cannula using a microinjection pump at a rate of 1 μl/min. The cannula was left in place for 5 min after completion of each injection and then slowly retracted. Equivalent injections of saline were made in control animals. Note that 30 min prior to surgery, rats received an intraperitoneal injection of desipramine hydrochloride (20 mg/kg, i.p., Sigma Co.) to protect noradrenergic neurons and fibers.

### Rotation Behavior Test

Two weeks after 6-OHDA injection, rats injected with 6-OHDA and control rats with saline injection were placed in a cylindrical container (300 mm diameter). Methamphetamine (3 mg/kg, i.p.) was injected to trigger rotational behavior. The rotational behavior was counted at 10-min intervals for 60 min after methamphetamine administration. Animals with >7 turns/min of rotational behavior were included in the study, as DA neurons and fibers of those rats are destroyed after 6-OHDA lesions (23).

### PAG Cannulation and Drug Infusion

Then, 3 days were allowed before the experiments. Rats were implanted with a stainless steel guide cannula (0.8 mm, o.d.) with sodium pentobarbital (60 mg/kg, i.p.), and then the guide cannula was secured to the skull. Stereotaxic coordinates for the dorsolateral PAG (dl-PAG) were 7.6 mm posterior to the bregma, 0.65 mm lateral to the midline, and 4.2 mm ventral to the brain surface.

Following this, cannula was connected to an osmotic minipump (Alzet pump brain infusion kit, DURECT Inc., Cupertino, CA, USA) with polycarbonate tubing. The pumps were placed subcutaneously between the scapulae and loaded with vehicle [artificial cerebrospinal fluid (aCSF)] as control or each PIC receptor antagonists, namely IL-1Ra (IL-1β receptor antagonist), SC144 (gp130 antagonist to block IL-6R), and etanercept (TNF-α receptor antagonist) (Tocris Co., Ellisville, MO, USA). In a subgroup, muscimol, agonist of GABAa receptors was loaded. The PIC receptor antagonists in 10 μM of concentration and muscimol in 100 μM of concentration were delivered at 0.25 μl/h (Alzet Model 1003D/3-day-delivery DURECT Inc., Cupertino, CA, USA). This intervention allowed animals to receive continuous PAG infusion *via* the osmotic minipumps before the experiments, and brain tissues were taken out. Note that all drugs were dissolved in aCSF as a final concentration.

### Pain Sensitivity

Rats were placed in individual plastic boxes to acclimate for >30 min in order to quantify the mechanical sensitivity of the hind paw. Mechanical withdrawal threshold (PWT) of rat hind paw responding to the stimulation of von Frey filaments was examined. A series of calibrated von Frey filaments (ranging from 0.5 to 18.0 g) were applied perpendicularly to the plantar surface of the hind paw with a sufficient force to bend the filaments for 60 s or until paw withdrew. If a response was seen, the filament of next lower force was given. Without a response, the filament of next greater force was used. To avoid injury during tests, the cutoff strength of the von Frey filament was 18 g. The tactile stimulus producing a 50% likelihood of withdrawal was determined using the “up-down” method (24). Each test was repeated twice at roughly 2 min intervals. The mean value was used as the force producing a withdrawal response.

Rat withdrawal latency (PWL) to a radiant heat was measured to examine thermal hyperalgesia. Rats were positioned separately in cages on an elevated glass platform and allowed for 30 min acclimation. Each hind paw received three stimuli with a 10 min interval, and the mean of the three withdrawal latencies was defined as PWL. The heat was maintained at a constant intensity. To prevent tissue damage, the cutoff latency was set at 20 s. All the behavioral tests were performed in a blind style.

At the end of the experiments, 2% Evans Blue in 0.25 μl was infused through the cannula. Then, the animals were anesthetized by sodium pentobarbital and intra-cardiacally perfused with physiological saline followed by 4% of paraformaldehyde solution. The midbrain was sectioned, and the location of injection sites was verified by histological examination of blue dye according to the atlas of Swanson (25). The rats were included for data analysis with microinjection site that was localized within the dl-PAG.

### ELISA Measurements

The rats were first euthanized by overdose sodium pentobarbital (120 mg/kg, i.p.), and then the dorsolateral regions of PAG were dissected under an anatomical microscope. Total protein of the PAG tissue was then extracted by homogenizing sample in ice-cold radioimmunoprecipitation assay buffer with protease inhibitor cocktail kit. The lysates were centrifuged, and the supernatants were collected for measurements of protein concentrations using a bicinchoninic acid assay reagent kit. The levels of IL-1β, IL-6, and TNF-α were examined using an ELISA assay kit (Promega Corp.) corresponding to the provided description and modification. Briefly, polystyrene 96-well microtiter immunoplates were coated with affinity-purified polyclonal rabbit anti-IL-1β, anti-IL-6, and anti-TNF-α antibodies. Parallel wells were coated with purified rabbit IgG for evaluation of non-specificity. After overnight incubation, the diluted samples and the PICs standard solutions were distributed in each plate. The plates were washed and incubated with anti-IL-1β, anti-IL-6, and anti-TNF-α galactosidase. Then, the plates were washed and incubated with substrate solution. After incubation, the optical density was determined using an ELISA reader. Note that measured absorbance on an ELISA plate reader was set at 450 and 550 nm and subtracted 550 nm values from 450 nm values to correct for optical imperfections in the microplate. If 550 nm was not available, it was measured at 450 nm only. In the similar way, the levels of GABA were determined (LDN Diagnostics, Inc. Colorado Springs, CO, USA) according to the provided description and modification.

### Western Blot Analysis

Similar to the ELISA, dl-PAG tissues were removed. In order to determine the expression of PIC receptors on cell surface, PAG tissues were incubated with Sulfo-NHS-LC-Biotin (1 mg/ml, Pierce) for 30 min on ice, as described previously (26). Because biotin is impermeable to the cell membrane, only proteins on the cell surface were biotinylated. The unbound biotin in the solution was removed by 5× wash of PAG tissues. PAG tissues were then homogenized and centrifuged at 13,500 × *g* (4°C) for 12 min. A sample (200 μg protein) was incubated with streptavidin beads (20 μl) for 3 h at 4°C. The beads were washed 3× with RIPA buffer, precipitated by centrifugation, and collected. Sample buffer (50 μl) was added to the collected beads and boiled for 3 min. Beads were pelleted again by centrifugation, and the supernatant was collected. The supernatant was diluted to the same volume as the starting material (i.e., 200 μg total protein). Total and membrane samples in equal volume were applied to SDS-PAGE. Membranes were incubated with the rabbit anti-IL-1R, anti-IL-6R, and anti-TNFR1primary antibodies (1:500, obtained from Neuromics and Abcam Co.). After being fully washed, the membrane was incubated with horseradish peroxidase-linked anti-rabbit secondary antibody (1:250) and visualized for immunoreactivity. The membrane was also processed to detect β-actin for equal loading. The bands recognized by the primary antibody were visualized by exposure of the membrane onto an X-ray film. The film was then scanned, and the optical densities of protein bands were analyzed using the Scion Image software. Then, values for densities of immunoreactive bands/β-actin band from the same lane were determined. Each of the values was then normalized to a control sample.

### Statistical Analysis

All data were analyzed using a one-way analysis of variance. As appropriate, Tukey’s *post hoc* analyses were utilized to determine differences between groups. Values were presented as means ± SE. For all analyses, differences were considered significant at *P* < 0.05. All statistical analyses were performed by using SPSS for Windows version 13.0 (SPSS Inc., Chicago, IL, USA).

## Results

### Levels of PICs and Expression of PIC Receptors

**Figure 1:**
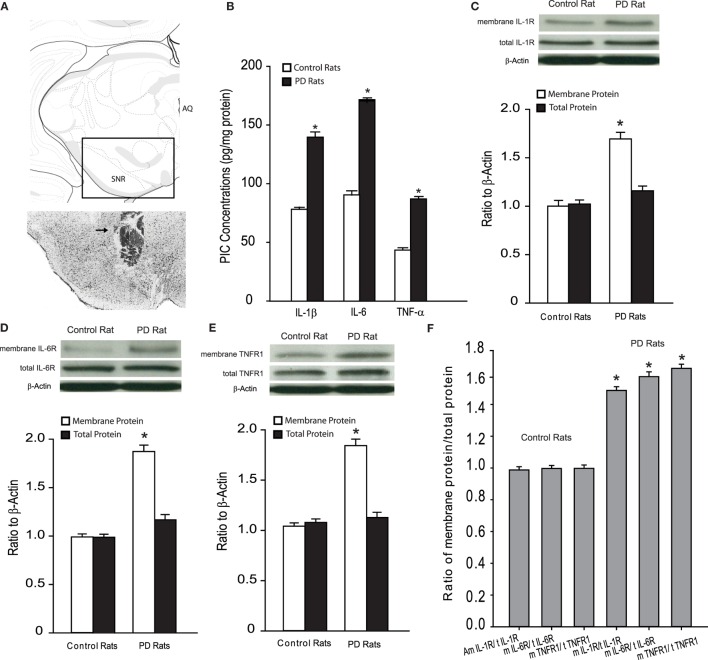
**(A)** Top panel: showing a rectangle area selected for the photograph of a histological section. Bottom panel: a histological section shows the location of injection cannula track. Arrow indicates cannula track. AQ, cerebral aqueduct; SNR, substantia nigra reticular part. **(B)** The levels of PICs in the dl-PAG. **(C–E)** The protein expression of PIC receptors (IL-1R, IL-6R, and TNFR1). Top panels are typical bands; bottom panels are averaged data in control rats and PD rats. Membrane PIC receptors are increased in PD rats, whereas total protein expression is not significantly altered. **(F)** The ratio of membrane PIC receptors protein/total PIC receptors protein. *P < 0.05 vs. control rats. The number of control rats = 15 and the number of PD rats = 20.

We examined the levels of PICs as well as total protein and membrane expression of PIC receptors in the dl-PAG of control rats (*n* = 15) and PD rats (*n* = 20). Figure 1A showed that IL-1β, IL-6, and TNF-α were elevated in PD rats as compared with control animals. Figures 1B–D demonstrated that the total PIC receptors expression in the PAG was not significantly altered in PD rats, but membrane PIC receptors expression was significantly increased in PD rats compared with control animals. Figure 1E further showed that the ratio of membrane and total PIC receptor densities was greater in the PAG of PD rats than that of control rats. Figure 1F shows that the ratio of membrane protein and total protein for IL-1R, IL-6R, and TNFR1 was 1.48 ± 0.15, 1.59 ± 0.18, and 1.67 ± 0.16 in PAG of PD rats (*P* < 0.05 vs. their respective controls).

### Pain Responses to Mechanical and Thermal Stimuli

**Figure 2:**
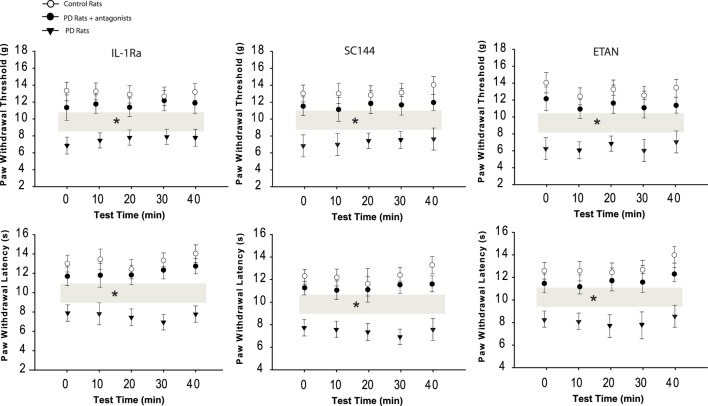
**Effects of blocking PIC receptors in the dl-PAG on pain responses to mechanical and thermal stimulation**. Mechanical and thermal hyperalgesia appeared lower in PD rats (*n* = 16) as compared with control animals (*n* = 10). Infusion of respective PIC receptor inhibitors into the PAG attenuated hypersensitive responses in PD rats (*n* = 12 in each group). *P < 0.05 vs. control rats and PD rats that received infusion of inhibitors over a 40-min testing time (indicated by gray bars).

Mechanical withdrawal threshold and PWL appeared to be less in PD animals (*n* = 16; *P* < 0.05 vs. control rats) as compared with control rats (*n* = 10). We further examined the effects of blocking PIC receptors (respective IL-1R, IL-6R, and TNFR1) in the dl-PAG on PWT and PWL in PD rats (*n* = 12 in each group). Figure 2 demonstrated that PWT and PWL were significantly increased during a 40-min period of the test with a 10-min interval after blocking each of PIC receptors (*P* < 0.05 vs. PD rats). Note that there were no differences in PWT and PWL between controls and PD rats with PIC receptors blocking (*P* > 0.05 vs. control rats). In addition, it is noted that blocking PIC receptors failed to significantly alter PWT and PWL in control animals.

### Engagement of GABA

**Figure 3:**
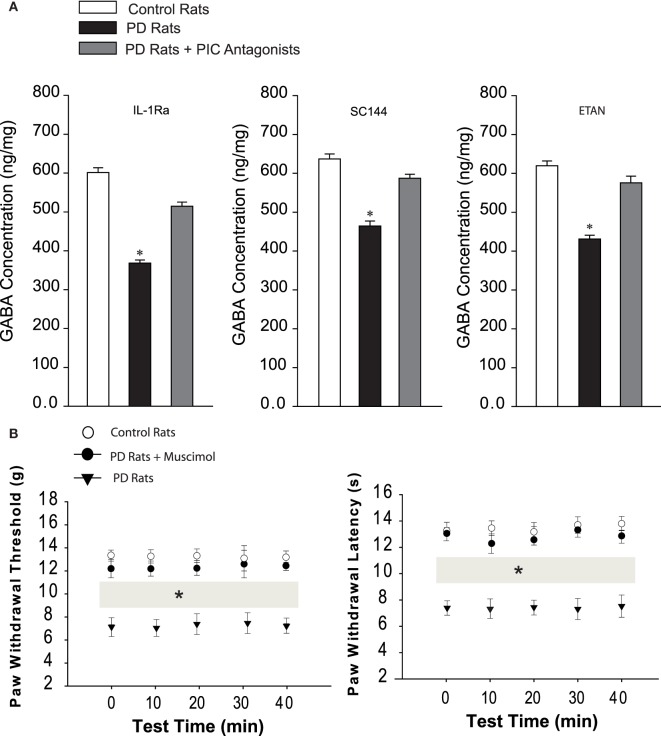
**(A)** The levels of GABA in the dl-PAG. The GABA was significantly diminished in PD rats (*n* = 12) as compared with control animals (*n* = 10). Injection of respective PIC receptor inhibitors largely restored impaired GABA. *P < 0.05 vs. control rats and rats with infusion of PIC receptor inhibitors (*n* = 8 in each group). **(B)** Effects of stimulation of GABAa receptors in the dl-PAG on pain responses to mechanical and thermal stimulation. Mechanical and thermal hyperalgesia appeared in PD rats (*n* = 10) as compared with control animals (*n* = 8). Infusion of GABAa receptor agonist, muscimol, into the PAG attenuated hypersensitive responses in PD rats (*n* = 12). *P < 0.05 vs. control rats and PD rats with infusion of muscimol over a 40-min testing time (indicated by gray bars).

Figure 3A demonstrated that the levels of GABA were significantly decreased in the dl-PAG of PD rats compared with control animals. The levels of GABA were 623 ± 21 ng/mg in control rats (*n* = 10) and 418 ± 18 ng/mg in PD rats (*n* = 12, *P* < 0.05 vs. control rats). With infusion of respective PIC receptor antagonists lessened, GABA was restored (*n* = 8 in each group, *P* < 0.05 vs. PD rats), but no significant differences were observed in GABA levels between control animals and PD animals with PIC receptors blocking (*P* > 0.05 vs. control rats).

We further examined the effects of stimulation of GABAa by infusion of muscimol in the dl-PAG on PWT and PWL in PD rats. Figure 3B showed that PWT and PWL were significantly increased during a 40-min period of the test with a 10-min interval after stimulation of GABAa (*P* < 0.05 vs. PD rats). Also, no differences in PWT and PWL were observed between controls and PD rats with muscimol (*P* > 0.05 vs. control rats). This result suggests the engagement of GABA in hypersensitive mechanical and thermal responses in PD rats.

## Discussion

Overall, the main findings of the present study are that (1) IL-1β, IL-6, and TNF-α and their receptors in membrane expression are upregulated in the dl-PAG of PD rats; and (2) blocking those individual receptors in this brain region attenuates hypersensitive responses to mechanical and thermal stimuli in PD rats likely by improving impaired GABAergic descending inhibitory system.

Nigrostriatal lesions induced by 6-OHDA in rats are widely used to study PD (27, 28). It has been reported that 6-OHDA injected into the medial forebrain bundle of rats to lead to extensive destruction of DA neurons of the substantia nigra pars compacta (29). A unilateral lesion of the nigrostriatal pathway also causes rotational behavior toward the lesioned side after administration of methamphetamine (30, 31). In the present study, the same rat model of PD was used, and consistent with prior studies, we have observed that rotational behavior appeared >7 turns/min after methamphetamine in 6-OHDA-injected rats.

Evidence has suggested that antinociception is mediated partly by descending pathways arising from the midbrain PAG (32, 33). Early studies showed that electrical stimulation or opioids microinjected into the PAG produced profound long-lasting antinociception (32, 33). In particular, activated neuronal cells are identified in PAG of PD rats evoked by 6-OHDA (31), suggesting that neural substrates are likely present within the PAG in engagement of the abnormalities in pain response observed in PD. Furthermore, previous studies showed that PIC mediators appear in the PAG, and activation of PICs in the PAG plays a role in modulating pain response or is involved in morphine withdrawal response (34, 35). Nonetheless, to the best of our knowledge, data of our present study have shown for the first time that PIC signal pathways in the PAG play a role in regulating abnormal pain response in a rat model of 6-OHDA-induced PD.

It is well-known that IL-1β is involved in the immune response and signal transduction both in the periphery and the central nervous system (36). IL-1β produced in the nervous system regulates the function of neuron and glia cells (37). Prior studies specifically demonstrated that IL-1β contributes to inflammatory and neuropathic pain (38). Increased level of IL-1β has been observed in the cerebrospinal fluid of chronic pain patients (39) and in the brainstem, contralateral thalamus/striatum, and prefrontal cortex of rats with spared nerve injury (40). A recent study showed that inhibition of melanocortin 4 receptor in the PAG not only blunts mechanical allodynia and thermal hyperalgesia but also delays the development of pain facilitation induced by peripheral nerve injury (41). This further decreases the expression of levels of IL-1β, IL-6, and TNF-α (41). Treatments with anti-IL-1β neutralizing antibodies or with IL-1β receptor antagonist (IL-1Ra) have also been reported to attenuate or block the hyperalgesia induced by a various nociceptive injuries (38, 42). Consistent with these prior findings, in the current study, we found that membrane expression of IL-1R was increased in the dl-PAG of PD rats, and blocking IL-1R in this brain region attenuated hypersensitive responses to mechanical and thermal stimuli in PD rats.

IL-6 complexes with membrane-bound or soluble IL-6R to stimulate cells expressing the signal transducer glycoprotein (gp130) (43, 44). Most cells are lacking of membrane-bound IL-6R and are thus unresponsive to IL-6. Nevertheless, they still react to IL-6 complexed with a soluble form of the IL-6R (sIL-6R) to activate gp130, a pathway called “trans-signaling” (44). Thus, in the current study, we used SC144, a gp130 inhibitor, to block IL-6-mediated signal transduction in order to examine engagement of the IL-6R in GABAergic signals and pain response thresholds to mechanical and thermal stimuli in PD rats.

The effects of TNF-α are *via* stimulation of two TNF-α receptor subtypes, TNFR1 and TNFR2 (45). TNFR1 is present entirely on neuronal cells and plays a functional role, whereas TNFR2 is located predominantly on macrophages and/or monocytes in response to inflammation. Thus, in our current study, application of ETAN lessens GABA in the PAG of PD rats and attenuates pain response, it is likely *via* TNFR1. In addition, we observed distinct expression of TNFR1 receptors in the PAG of PD rats.

Microglia are one of the major cell types involved in the inflammatory responses in the central nervous system (46). The evidence from a prior study revealed reactive microglia in the substantia nigra pars compacta of human postmortem brain tissue, suggesting the involvement of neuroinflammation in PD pathogenesis (47). Prior reports using positron emission tomography (PET) have further indicated that there are amplified activities of microglia in various brain regions of PD patients (12, 14). Moreover, activation of microglia in the substantia nigra pars compacta and striatum is amplified in various types of PD animal models (13, 48). Biochemical analysis also showed exaggerated pro-inflammatory mediators, including IL-1β and TNF-α, in the midbrain of PD patients (49). These data indicate the involvement of immune mechanisms in PD pathophysiological process.

In addition, prior studies showed that astrocytes play an important role in regulating the neuroinflammatory response in PD. Similar to microglia, astrocytes react to the inflammatory stimulations by producing PICs, including IL-1β and TNF-α, both *in vitro* and *in vivo* (46, 50). Reactive astrogliosis with the increased glial fibrillary acidic protein, hypertrophy of cell body and cell extensions has been found in PD animal models (51).

In the present study, we demonstrated that cell membrane PIC receptors are upregulated in the dl-PAG of PD rats. However, the total protein expression of PIC receptors was not considerably altered in the PAG of PD rats, indicating that PIC receptors trafficking to the cell membrane of PAG is particularly amplified in PD rats (26). The underlying mechanism for the increase in trafficking of PIC receptors following 6-OHDA lesions needs to be determined. The elevated PICs were also observed in PD rats in the present study. Accordingly, we assume that PICs are likely released from the glial cells, and this signal is likely to lead to upregulation of membrane PIC receptors. Nevertheless, it is speculated that the increased activities in the PIC pathways are likely to result in neuronal loss within the PAG since PICs is engaged in the process of apoptosis, which has been observed in PD brains (52).

Interestingly, results of our present study further demonstrated that the levels of GABA were significantly decreased in the dl-PAG of PD rats. Prior studies showed antinociceptive effects evoked by stimulation of this region of PAG (7, 9). This supports our hypothesis that activation of PIC receptors within the PAG plays a de-inhibitory role in regulating the descending pain pathways. When PIC receptors are blocked in the dl-PAG, the abnormal descending pain pathways are largely restored because we have observed that chronic infusion of PIC antagonists lessened amplified pain responses in PD animals accompanied with increasing GABA levels in the PAG. Furthermore, our results found that mechanical and thermal hyperalgesia in PD rats was attenuated following stimulation of GABAa receptors in the dl-PAG by infusion of muscimol, suggesting that PIC receptors influence GABAergic transmission within this region of PAG and thereby amplify pain response.

We speculate that there are some possibilities that PICs and/or activation of PIC receptors can alter GABAergic pathway (53). (1) PICs inhibit the releases of GABA in the dl-PAG since a lower level of GABA was observed in this region in our current study; (2) the increased PICs are likely to damage neurons of the dl-PAG thereby leading to a reduction in GABA; (3) the amplified membrane PIC receptors observed in our study are likely to modulate activities of ion channels on neurons of the dl-PAG and decrease the GABA levels or attenuate GABAergic transmission. Additional studies need to determine a precise mechanism responsible for how PICs alter GABAergic pathway within the dl-PAG in involvement of abnormal pain responses in PD.

### Study Limitations

In the current study, we examined the levels of PICs and their respective receptors in the PAG of control rats and rats injected with 6-OHDA, and then examined if blocking PIC receptors affected pain response and the levels of GABA. Our results showed that PIC pathway in the PAG of 6-OHDA-rats was upregulated by using Western Blotting and ELISA, but we did not perform the immunohistochemical experiment to indicate this result. We assumed that the effects of 6-OHDA on GABA were likely indirect *via* PICs signal. In addition, the previous studies have demonstrated that dopamine levels were decreased in SNc by 6-OHDA (31). Thus, we did not perform the immunohistochemical experiment to indicate this result in our current study. We must knowledge that those issues should be considered as study limitations, and adding this line of results obtained by immunohistochemical experiment would strengthen our current findings.

In conclusion, we have shown that the membrane PIC receptors are increased in the dl-PAG of PD rats and thereby de-inhibit GABAergic mediated-descending regulation in pain transmission. These abnormalities are likely to contribute to the development of mechanical and thermal hypersensitivity in PD animals. Results of this study provided a base for the mechanisms responsible for PD-induced pain. This further offers promising clues to target central nerve system for the development of new therapeutic strategies for managing intractable pain response in PD patients.

## Author Contributions

All authors listed, have made substantial, direct, and intellectual contribution to the work and approved it for publication.

## Conflict of Interest Statement

The authors declare that the research was conducted in the absence of any commercial or financial relationships that could be construed as a potential conflict of interest.
